# Quality of integrated female oncofertility care is suboptimal: A patient‐reported measurement

**DOI:** 10.1002/cam4.5149

**Published:** 2022-08-29

**Authors:** Michelle van den Berg, Suzanne E. J. Kaal, Teska N. Schuurman, Didi D. M. Braat, Caroline M. P. W. Mandigers, Jolien Tol, Jacqueline M. Tromp, Maurice J. D. L. van der Vorst, Catharina C. M. Beerendonk, Rosella P. M. G. Hermens

**Affiliations:** ^1^ Department of Obstetrics and Gynecology Radboud University Medical Center Nijmegen the Netherlands; ^2^ Department of Medical Oncology Radboud University Medical Center Nijmegen the Netherlands; ^3^ Dutch AYA ‘Young and Cancer’ Care Network IKNL Utrecht the Netherlands; ^4^ Center for Gynecologic Oncology Amsterdam The Netherlands Cancer Institute‐Antoni van Leeuwenhoek Hospital Amsterdam The Netherlands; ^5^ Department of Medical Oncology, Canisius‐Wilhelmina Hospital Nijmegen The Netherlands; ^6^ Department of Medical Oncology, Jeroen Bosch Hospital Den Bosch The Netherlands; ^7^ Department of Medical Oncology Amsterdam University Medical Center Amsterdam The Netherlands; ^8^ Department of Medical Oncology, Rijnstate Hospital Arnhem The Netherlands; ^9^ Department of IQ Healthcare Radboud University Medical Center Nijmegen the Netherlands

**Keywords:** cancer, female cancer patients, oncofertility care, quality indicators, quality of care

## Abstract

**Background:**

Clinical practice guidelines recommend to inform female cancer patients about their infertility risks due to cancer treatment. Unfortunately, it seems that guideline adherence is suboptimal. In order to improve quality of integrated female oncofertility care, a systematic assessment of current practice is necessary.

**Methods:**

A multicenter cross‐sectional survey study in which a set of systematically developed quality indicators was processed, was conducted among female cancer patients (diagnosed in 2016/2017). These indicators represented all domains in oncofertility care; risk communication, referral, counseling, and decision‐making. Indicator scores were calculated, and determinants were assessed by multilevel multivariate analyses.

**Results:**

One hundred twenty‐one out of 344 female cancer patients participated. Eight out of 11 indicators scored below 90% adherence. Of all patients, 72.7% was informed about their infertility, 51.2% was offered a referral, with 18.8% all aspects were discussed in counseling, and 35.5% received written and/or digital information. Patient's age, strength of wish to conceive, time before cancer treatment, and type of healthcare provider significantly influenced the scores of three indicators.

**Conclusions:**

Current quality of female oncofertility care is far from optimal. Therefore, improvement is needed. To achieve this, improvement strategies that are tailored to the identified determinants and to guideline‐specific barriers should be developed.

## Graphical abstract

This study demonstrated that the quality of integrated female oncofertility care measured with a set of systematically developed quality indicators is far from optimal and needs improvement.

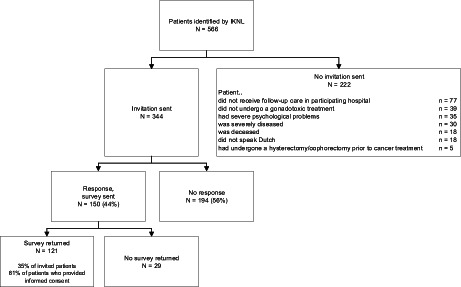

## 1 INTRODUCTION

The potential loss of fertility due to gonadotoxic cancer treatments is one of the most important concerns in female adolescent and young adult (AYA) cancer patients.^1^, ^2^, ^3^ Therefore, they are interested in receiving information about infertility risks and fertility preservation (FP) options.^4^, ^5^, ^6^ Current clinical guidelines recommend oncological healthcare professionals to provide information on infertility risks and FP options, and, if desired, to offer a referral to and counseling by a fertility specialist.^7^, ^8^, ^9^ Providing information to female AYA cancer patients affects quality of life positively, reduces long‐term regret, and reduces concerns regarding fertility.^1^, ^10^, ^11^ Despite these guidelines and positive effects, studies have shown that the proportion of patients provided with information on their infertility risks and FP options is suboptimal and varies greatly from 26% to 95%.^12^, ^13^, ^14^, ^15^, ^16^, ^17^ The referral process to a fertility specialist also shows variation in practice; only 9.8% to 67% is referred.^13^, ^15^, ^16^, ^18^

These low rates indicate a suboptimal adherence to female oncofertility guidelines and suggest a suboptimal quality of care. In order to improve quality of integrated female oncofertility care, a systematic assessment of current practice is necessary, just as an assessment of determinants that influence guideline adherence.^19^ Regarding the systematic assessment of current practice, most studies did not assess the quality of care systematically. They reported the number of FP discussions and referrals based on self‐reported practices by healthcare providers or medical record documentation. Both methods have limitations. Regarding self‐report, healthcare providers might overestimate their performance, and thus, a self‐report bias should be taken into account.^20^ Regarding medical record documentation, disparity between discussions and documentation exists, varying from 4% to 23%.^21^, ^22^ Therefore, it would be better to measure quality of integrated care by consulting actual female cancer patients, because it is more important to know what patients remember than what is documented in incomplete medical records. Furthermore, studies have shown that patients can accurately report on their cancer diagnosis, treatment, and characteristics.^22^, ^23^

To systematically assess the current quality of female oncofertility care, quality indicators (QIs) can be used. QIs are measurable elements of practice performance for which there is evidence or consensus that it can be used to assess quality, and hence change the quality of care provided.^24^ In our previous study, key recommendations for high‐quality integrated female oncofertility care were selected by means of a Delphi procedure with a multidisciplinary national expert panel.^25^ After translating these key recommendations into QIs, these are well suited to systematically assess the quality of integrated oncofertility care. Regarding determinants that influence guideline adherence, these can be found on patient, professional and hospital level. Insight into these determinants can explain the variation in care and should be taken into account when developing tailored improvement strategies to improve quality of care, since quality of care does not improve by itself.

In our current study, the first aim was therefore to systematically assess the quality of integrated female oncofertility care by a patient‐reported measurement of QIs. The second aim was to measure which determinants were associated with this quality of care to be able to develop tailored improvement strategies to improve quality of integrated female oncofertility care.

## 2 METHODS

### 2.1 Design and setting

This multicenter cross‐sectional study was conducted by means of a survey to female AYA cancer patients in six hospitals: three academic hospitals and three (large) non‐academic hospitals. In the Netherlands, patients receive multidisciplinary oncological care and can be referred for FP counseling by any medical specialist involved. FP counseling is performed by reproductive gynecologists. In the Netherlands, patients have no financial reasons to refrain from FP counseling or undergoing FP treatment, because it is covered by basic health insurance or by the hospital cryobanks themselves. This study was approved by the Medical Ethics Committee of Arnhem‐Nijmegen (NL61570.091.17) and was performed in accordance with the Declaration of Helsinki.

### 2.2 Study population

Female AYA cancer patients (18 up to and including 40 years) who were diagnosed in 2016 or 2017 and received a (potential) gonadotoxic treatment were eligible to participate. The Netherlands Cancer Registry was used by the Netherlands Comprehensive Cancer Organization (IKNL) to identify potentially eligible patients in each participating hospital. After identification, the patient's primary oncological healthcare provider was asked to assess the following exclusion criteria: Patient was deceased, severely diseased, had severe psychological problems, had undergone a hysterectomy and/or oophorectomy prior to the start of her cancer treatment, did not receive follow‐up care in the participating hospital, or did not understand the Dutch language.

### 2.3 Survey development

The survey was developed within our research team. The survey consisted of baseline and clinical characteristics (patient's age, partner relationship, diagnosis and cancer treatment, strength of wish to conceive) and questions that represented the QIs (see below). After reaching consensus with the research team, the survey was pilot‐tested in five female cancer survivors and four non‐cancer patients after which small adjustments were made.

### 2.4 Quality indicators

**FIGURE 1:**
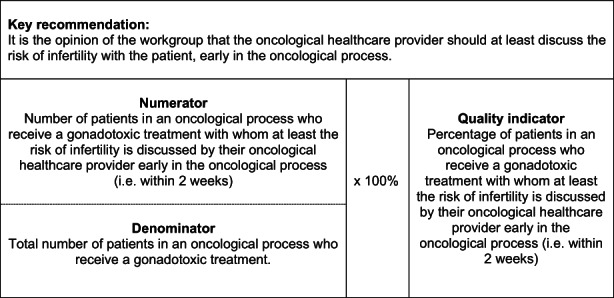
Example of quality indicator as a tool to measure quality of care.

In our previous research, we used a systematic Delphi procedure to select key recommendations for high‐quality integrated female oncofertility care with a multidisciplinary oncofertility expert panel including female cancer survivors.^25^ These key recommendations were first extracted from high‐quality international clinical guidelines on this subject and then selected and approved by the expert panel by means of three consensus rounds.^25^, ^26^ This resulted in 11 key recommendations. Subsequently, the research team transcribed the key recommendations into 11 QIs (defining numerators and denominators). An example of the transcription from key recommendation to a QI is shown in Figure 1. The questions that were developed to measure this QI were as follows: “Did your oncological healthcare provider discuss the risk of infertility due to the cancer treatment with you?” and “When did the oncological caregiver discuss the risk of infertility with you?”. A total of 40 questions were developed to measure the 11 indicators. The questions measured quality of female oncofertility care for female AYA cancer patients focusing on risk communication, referral, counseling, and decision‐making. All QIs are shown in Table 1.

**TABLE 1:** Quality indicators distributed over the domains in female oncofertility care

Domain	Quality indicators
Risk communication QI1 QI2 QI3	Percentage of patients in an oncological process who receive a gonadotoxic treatment…
	With whom at least the risk of infertility is discussed by their oncological healthcare provider early in the oncological process (i.e., within 2 weeks).
	With whom the consequences (of the treatment) for their fertility are discussed when they have a wish to conceive, and when their ovaries, uterus, hypothalamus, and pituitary gland (or all) are in the irradiation area (pelvic, skull, craniospinal irradiation, or total body irradiation).
	Of whom the oncological healthcare provider consulted an expert colleague when he/she had insufficient knowledge about fertility preservation.
Referral QI4	Percentage of patients in an oncological process who receive a gonadotoxic treatment to whom the opportunity of counseling with a gynecologist with expertise in fertility preservation is offered.
Counseling	Percentage of patients in an oncological process who receive a gonadotoxic treatment…
QI5	For whom an individualized selection and risk analysis has been executed within an expert multidisciplinary team (i.e., primary oncological healthcare provider and treating gynecologist).
QI6	(Who are estimated as medically fit for the procedure, are expected to be able to tolerate the treatment regimen, have sufficient time before the commencement of their cancer treatment, and are informed of the potential risks of hormonal treatment including the risks of cancer progression), with whom oocyte and embryo cryopreservation has been discussed during fertility preservation counseling.
QI7	To whom embryo cryopreservation is offered as an effective and safe method, when time and circumstances allow for it.
QI8	Who have had fertility preservation counseling with a gynecologist in which all aspects (a – h) have been discussed. a. The chance to preserve ovarian, uterine function, or both, and the chance of spontaneous pregnancy after cancer treatment. b. The chance to preserve ovarian, uterine function, or both, and the chance of pregnancy when using different fertility preservation methods, and expectations for the future. c. The risks of fertility preservation procedures: delay of cancer treatment, surgery (laparoscopy, laparotomy), risk of reintroducing the tumor (metastases) after autotransplantation of cryopreserved ovarian tissue, premature menopause after cancer treatment and unilateral and partial oophorectomy. d. The conditions to undergo fertility treatment after cancer treatment, (number of years a patient should be relapse‐free after curation, posthumous reproduction, etc.). The contracts should also be discussed. e. Alternatives, such as oocyte donation, gestational surrogacy, or adoption. f. Necessary tests before a fertility preservation treatment, such as standard screening for viral pathogens and sexually transmitted diseases. g. Hormonal screening through blood testing. h. Possibilities to treat endocrine consequences owing to the loss of ovarian function.
QI9	Who have been well‐informed about all aspects of the treatment prior to performing emergency IVF.
Decision‐making QI10 QI11	Percentage of patients in an oncological process who receive a gonadotoxic treatment…
	With whom a shared decision has been made concerning protecting future fertility (together with oncological healthcare provider and gynecologist)
	Who have had fertility preservation counseling which was supported with written, digital information, or both.

### 2.5 Data collection

Eligible patients received an invitation letter from their primary oncological healthcare provider to participate. After obtaining informed consent, a paper version of the survey was sent by mail in 2020/2021. In total, 344 patients were invited to participate. If no informed consent or completed survey was received within 3 weeks, one reminder was sent.

### 2.6 Data analysis

QI adherence scores were calculated per indicator. Herewith, current practice was expressed as the percentage of patients who received care as recommended in the QI. In addition, hospital variation was calculated.

To evaluate which determinants were associated with the quality of integrated female oncofertility care, we first studied the univariate relation between indicator scores (dependent variables) and determinants (independent variables) that could influence the indicator score. Determinants were extracted from literature and included age, relationship status, parity, strength of wish to conceive, type of cancer, type of cancer treatment, time before start of cancer treatment, type of healthcare provider, and type of hospital.^12^, ^15^, ^16^, ^18^, ^27^, ^28^ We used a generalized linear mixed model which accounts for the nested structure of data, because individual patients (patient level = 1) are nested within hospitals (hospital level = 2). For determinants with *p* < 0.20 in univariate analyses, we performed multivariate logistic regression. Collinearity between determinants was also tested. If a correlation (>0.6) was detected, the most relevant variable with respect to content was included in the multivariate analyses. Odds ratios described the association between indicator scores and determinants. Indicator scores were recalculated for each indicator and for each part of the associated determinant. For example, an indicator could be recalculated for all patients aged 18–29 years and for all patients aged 30–41 years.

## 3 RESULTS

### 3.1 Study population

**FIGURE 2:**
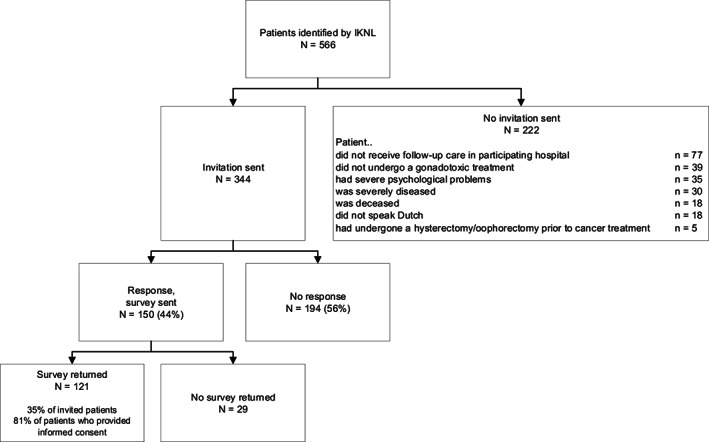
Flowchart: eligibility and response.

In total, 566 patients were identified by IKNL of whom 344 met the inclusion criteria. Figure 2 shows reasons for exclusion. A total of 121 out of 344 (35%) surveys were returned. Mean patient age at diagnosis was 34 years, 60.3% was diagnosed with breast cancer, and 37.2% had undergone FP treatment (Table 2).

**TABLE 2:** Baseline characteristics

Characteristic	Number	%
Response rate total and per hospital Academic hospital Hospital A Hospital B Hospital C Large non‐academic hospital Hospital D Hospital E Hospital F	121/344 24/56 15/55 50/147 11/26 11/33 10/27	35 42.9 27.3 34 42.3 33.3 37
Mean age at diagnosis	34.0 years (range 18–41, SD 5.1)	
Type of cancer Bladder cancer Brain tumor Breast cancer Cervical cancer Colorectal cancer Head and neck cancer Leukemia Lung cancer Lymphoma Osteosarcoma Ovarian cancer Soft tissue sarcoma	1 2 73 14 5 1 3 1 15 2 2 2	0.8 1.7 60.3 11.6 4.1 0.8 2.5 0.8 12.4 1.7 1.7 1.7
Cancer treatment Breast surgery Chemotherapy Hormone therapy Immunotherapy Irradiation therapy Breast Skull/Craniospinal Pelvic Total body Uterine or ovarian surgery	72 112 45 20 80 56 8 10 6 11	59.5 92.6 37.2 16.5 66.1 70 10 12.5 7.5 9.1
Relationship status Yes, more than 2 years Yes, less than 2 years No, single	90 20 11	74.4 16.5 9.1
Parity Nulliparous Parous Para 1 Para 2 Para 3 Para 4	50 71 29 29 12 1	41.3 58.7 40.8 40.8 17 1.4
Strength of wish to conceive at diagnosis (mean, scale 1–10) Nulliparous Parous	5.8 (range 1–10, SD 3.5, median 5) 7.0 (range 1–10, SD 2.9, median 7.5) 4.9 (range 1–10, SD 3.6, median 5)	
Number of patients who had undergone fertility preservation treatment (3 patients had 2 treatments) Oocyte cryopreservation Embryo cryopreservation Ovarian tissue cryopreservation Trachelectomy or unilateral ovariectomy GnRH agonists	45/121 24 11 3 9 1	37.2 53.3 24.4 6.7 20 2.2

### 3.2 Current quality of female oncofertility care and variation

Guideline adherence, measured with the 11 QIs, is presented in Table 3. Overall, 8 out of 11 QIs scored below 90% adherence. Regarding the different domains, for the domain risk communication a score of 72.7% was reported for “discussing the risk of infertility within 2 weeks after diagnosis” (QI1). The indicator with the lowest score in this domain was “consulting an expert colleague when an oncological healthcare provider has insufficient knowledge” (QI3, 43.8%); the indicator with the highest score was “to discuss infertility risks when a patient receives gonadotoxic radiation therapy” (QI2, 95.8%). In the referral domain, the indicator “offering FP counseling with a gynecologist to a patient” scored 51.2% (QI4). Indicators in the counseling domain showed a range from 18.8 to 100%, with “discussing all aspects in FP counseling” (QI9) as lowest score, and “discussing oocyte and embryo cryopreservation with eligible patients” (QI6) as highest score. In the domain of decision‐making, a score of 73.3% was reported for “making a shared decision” (QI10) and a score of 35.5% for “supporting the decision with written and/or digital information” (QI11).

**TABLE 3:** Results of quality indicators, variation per hospital and determinants

Quality indicator	Total score Nominator/denominator	%	Variation per hospital %	Missing data %	Determinants (in multivariate analyses)
QI1—Risk of infertility discussed within 2 weeks after diagnosis At diagnosis Within 1 week Within 2 weeks After 2 weeks Not discussed	88/121 29 35 24 27 6	72.7	46.7–83.3	0	None
QI2—Risk of infertility discussed when pelvic, skull, craniospinal, or total body irradiation takes place	23/24	95.8	85.7–100	0	None
QI3—Oncological healthcare provider consulted expert colleague when he/she had insufficient knowledge about fertility preservation Insufficient knowledge Consulted expert colleague	7/16 22/120 7/16	43.8	25–100	27.3	None
QI4—Opportunity of counseling with a gynecologist is offered	62/121	51.2	40–70.8	0	Patient's age; Strength of wish to conceive
QI5—Individualized selection and risk analysis has been executed within an expert multidisciplinary team prior to fertility preservation treatment Selection and risk analysis Multidisciplinary team	19/21 28/35 19/21	90.5	0–100	53.3	None
QI6—Oocyte and embryo cryopreservation has been discussed with eligible patients Medically fit Tolerate fertility preservation treatment Sufficient time Risks hormone treatment	22/22 33/36 36/38 37/39 22/22	100	100	47.5	None
QI7—Embryo cryopreservation is offered as an effective and safe method, when time and circumstances allow for it	34/39	87.2	75–100	2.6	None
QI8—All aspects (a – h) have been discussed in fertility preservation counseling a. Chance preserve fertility after cancer b. Chance preserve fertility after FP c. Risks FP treatment d. Conditions to have FP treatment e. Alternative family building options f. Necessary tests before FP treatment g. Hormonal screening h. Possibilities to treat endocrine consequences	6/32 28/37 20/31 7/26 16/34 14/41 27/37 6/32 12/37	18.8	0–40	47.5	None
QI9—Well‐informed about all aspects of the treatment prior to performing emergency IVF	27/33	81.8	60–100	5.7	None
QI10—Shared decision has been made concerning protecting future fertility	44/60	73.3	42.9–100	0	Type of health care provider
QI11—Decision was supported with written and/or digital information	43/121	35.5	6.7–50	0	Strength of wish to conceive; Time before start of cancer treatment

In all four domains, QI scores varied greatly among the six participating hospitals (Table 3). In 10 out of 11 QIs >20% variation was observed among hospitals.

### 3.3 Determinants

Results from univariate analyses are shown in Table S1; for determinants with *p* < 0.20, multivariate analyses were performed. Multivariate analyses showed that 3 determinants at patient level (patient's age, strength of wish to conceive, and time before start of cancer treatment) and 1 determinant at professional level (type of healthcare provider) influenced the scores of 3 indicators significantly (Table 4). Patients who were younger (<30 years), or with a higher wish to conceive (score 6–10) showed a greater adherence to the indicator “offering FP counseling with a gynecologist” (QI4). Treatment by a medical oncologist was associated with a lower score for “making a shared decision” (QI10), and patients with a higher wish to conceive or with more time before start of cancer treatment (>2 weeks) showed a higher score for “receiving written and/or digital information” (QI11).

**TABLE 4:** Determinants in multilevel analyses and stratified indicator score

Quality indicator	Determinants	*N*	Multivariate analysis Odds ratio (95% CI), *p*‐value	Stratified indicator score %
QI4—Opportunity of counseling with a gynecologist is offered	Patient's age 18–29 years 30–41 years Strength of wish to conceive 1–5 6–10	24 97 61 60	4.03 (1.34–12.23), p0.015 Ref Ref 3.18 (1.46–6.95), p0.004	79.2% 44.3% 36.1% 66.7%
QI10—Shared decision has been made concerning protecting future fertility	Type of healthcare provider Medical oncologist Other than medical oncologist	21 39	Ref 5.22 (1.29–21.12), p0.02	52.4% 84.6%
QI11—Decision was supported with written and/or digital information	Strength of wish to conceive 1–5 6–10 Time before start cancer treatment <2 weeks >2 weeks	61 60 29 92	Ref 2.69 (1.20–6.05), p0.02 Ref 3.03 (1.07–8.62) p0.04	26.2% 45% 20.7% 40.2%

## 4 DISCUSSION

This study demonstrated that the quality of integrated female oncofertility care measured by a patient‐reported measurement with a set of systematically developed QIs is far from optimal. Improvement potential (indicators with <90% adherence) was found for 8 out of the 11 QIs representing all domains in female oncofertility care: risk communication, referral, counseling, and decision‐making. In addition, a great variation in indicator scores (>20% for 10 out of 11 QIs) was seen among hospitals. Four determinants (patient's age, strength of wish to conceive, time before cancer treatment, and type of healthcare provider) were found to significantly influence the scores of three indicators on referral and (support of) shared decision‐making.

To the best of our knowledge, this is the first study in which the quality of integrated female oncofertility care was systematically assessed with a set of QIs, using a patient‐reported measurement. In a previous study conducted in the Netherlands, oncological healthcare providers were asked via a survey how often they discuss fertility issues with female cancer patients and refer them for FP counseling. In total, 79% usually or always discussed fertility issues, and 54% usually or always referred patients for FP counseling.^15^ These percentages are in line with our indicator scores on these items (QI1 72.7% and QI4 51.2%) meaning that still no improvement in quality of care has taken place.

Comparing with patient survey studies in other countries without a systematic assessment, our indicator scores are lower. An Australian study reported a discussion rate about infertility risks of 88%, and referral rate of 59%,^12^ and an American survey study found a discussion rate of 74.5%.^29^ This could be explained by the study population; in those studies, patients were much younger (15 and 21 years, respectively), which was a determinant we found to increase referral rates.

Furthermore, in contrast to our study, these previous studies focused on one or two elements of female oncofertility care, for example on receiving information, receiving fertility preservation counseling, or having fertility preservation treatment. We focused on all domains in female oncofertility care, particularly on information provision, on offering referral for counseling, on counseling by a gynecologist, and on fertility preservation decision‐making. All these domains have shown to be important in delivering high‐quality integrated female oncofertility care as these were selected as key elements by a multidisciplinary expert panel and based on international evidence‐based guidelines.^25^ In addition, patients want to be fully informed about all aspects of fertility preservation and supported in their decision.^4^, ^30^, ^31^ Unfortunately, we found low scores for the indicators that cover these items; about one fifth for discussing all aspects in FP counseling, one third for supporting the decision with written and/or digital information, and three quarters who have made a shared decision regarding FP. This underlines the fact that not only discussion and referral rates need improvement, but also FP counseling and FP decision‐making. A way to improve FP counseling and decision‐making could be the provision of a decision aid.^32^, ^33^ We developed a FP decision aid that is tailored to cancer type and associated cancer treatments.^34^ First results are promising, but future research should evaluate its effectiveness.

We found that a higher patient's age was associated with lower referral rates. This is in line with previous studies.^35^, ^36^, ^37^ This might be explained by the fact that healthcare providers think that patients who are older do not have a wish to conceive anymore, without asking for it. Furthermore, we found that a higher wish to conceive was associated with higher referral rates and receiving written and/or digital information. A possible explanation is that these patients might ask themselves for a referral. Both associations underline the importance of asking a patient for her needs and wishes regarding future fertility. Our determinants could be used to develop tailored improvement strategies to improve quality of integrated female oncofertility care. For example, oncology nurses can play an essential role in this, as they already play an important role in cancer care programs, and as patient advocates.^38^, ^39^, ^40^

A strength of our study is that it is the first study that systematically assessed the quality of integrated female oncofertility care using QIs extracted from high‐quality international clinical guidelines and using a patient‐reported measurement. Furthermore, we included a diverse study population, that is, female cancer survivors with all types of cancers and treatments, from all types of hospitals, of all reproductive ages, with and without children or relationship, and with a variety in their strength of wish to conceive. This ensured that our results represent clinical practice and are generalizable to other countries.

However, despite the systematic assessment, some limitations should be considered in the interpretation of our results. Selection bias could have occurred because of our low response rate, although this is in line with other survey studies among AYA patients,^41^ and because each primary oncological healthcare provider had to give consent to invite a patient for participation. In case of exclusion, the reason for exclusion was shared, however, this could not be checked by the research team because of regulations regarding data protection. This could have led to exclusion of patients that did not adhere to QIs, while they did match the inclusion criteria which would mean the quality of care was even lower. We have tried to minimize this selection bias by explicitly asking each healthcare provider to stick to the in‐ and exclusion criteria, and in doubt, to discuss the case with the research team. In addition, bias could have occurred because of selective response by patients. For example, dissatisfied patients might be more likely to complete the survey leading to an underestimation of quality of care. However, in comparison with other studies, we found a high rate of patients who had FP treatment (37%).^11^, ^42^ This may indicate that patients who have received fertility preservation care as recommended were more likely to participate than patients who did not receive recommended care. This might have led to an overestimation of the quality of integrated female oncofertility care, and in fact, the quality was even lower. Furthermore, recall bias could have played a role because patients might have a poor recall on discussions about fertility issues when they are confronted with a cancer diagnosis and because they were asked to fill in questions three to 4 years after their diagnosis, treatment, and consultation. However, it is also known from other qualitative studies among cancer survivors that fertility issues are almost as important as the devastating cancer diagnosis for many patients.^3^, ^43^ This was confirmed in our study, as for the “basic” questions about fertility discussions (QI1, 2, 4, 10, 11) there were no missing data. Regarding the questions that asked for very specific details of information given in consultations (QI6 and 8), or that asked for things a patient might not know (QI3 and 5) relatively many data were missing. So, in future it should be evaluated if these QIs could be measured in medical records or provided by healthcare providers to minimize this bias. Last, it might be interesting to compare the survey results to what is documented in the medical record to check for the disparity between what patients remember from the discussion and the documentation.

In conclusion, our study showed a considerable difference between daily practice performance and care as described in evidence‐based guidelines. Low guideline adherence is associated with lower, and therefore suboptimal, quality of care since guidelines assist in delivering the most optimal care. Suboptimal care increases concerns regarding fertility and long‐term regret, affecting female cancer AYA patients' quality of life negatively.^2^, ^10^, ^11^ Therefore, improvement is needed. To achieve this, improvement strategies that are tailored to the identified determinants and to guideline‐specific barriers should be developed, for example a decision aid.^34^, ^44^, ^45^ Our QI set could then be used to evaluate whether the improvement strategy has a positive effect on quality of integrated female oncofertility care in both the Netherlands and internationally.

## AUTHOR CONTRIBUTIONS

MvdB, DDMB, CCMB, and RPMGH involved in study concept and design. MvdB, CCMB, SEJK, TNS, CMPWM, JT, JMT, and MJDLvdV involved in data acquisition. MvdB, DDMB, CCMB, and RPMGH involved in data analysis and interpretation. MvdB involved in writing original draft. All authors involved in reviewing the article and final approval of article.

## FUNDING INFORMATION

This research was funded by the Paul Speth Fund and the Radboud Oncology Fund.

## CONFLICT OF INTEREST

The authors declare no conflict of interest.

## ETHICS APPROVAL AND CONSENT TO PARTICIPATE

This study was approved by the Medical Ethics Committee of Arnhem‐Nijmegen (NL61570.091.17) and was performed in accordance with the Declaration of Helsinki. Written consent to participate was obtained for all participants.

## Supporting information


Table S1

## Data Availability

Study data are available upon request.
