# Supplementary material for: Quality of integrated female oncofertility care is suboptimal: A patient‐reported measurement
Source: Cancer Med. 2022 Aug 29;12(3):2691–701. doi: 10.1002/cam4.5149 (PMC9939180; doi:10.1002/cam4.5149)
Supplement: Supplementary file 1 — Table S1 [file CAM4-12-2691-s001.docx]

**Supplementary Table 1. Determinants in univariate analyses**

|  | **QI1** - Risk of infertility is discussed within 2 weeks  p-value | **QI4** - Opportunity of counseling with a gynecologist is offered  p-value | **QI10** - Shared decision has been made concerning protecting future fertility  p-value | **QI11** - Decision was supported with written and/or digital information  p-value |
| --- | --- | --- | --- | --- |
| **Patient’s age** | 0,812 | 0,006* | 0,186* | 0,239 |
| **Relationship status** | 0,489 | 0,444 | 0,476 | 0,941 |
| **Parity** | 0,110* | 0,001* | 0,560 | 0,237 |
| **Strength of wish to conceive** | 0,534 | 0,002* | 0,082* | 0,026* |
| **Type of cancer** | 0,754 | 0,973 | 0,610 | 0,119* |
| **Type of cancer treatment** | 0,076* | 0,468 | 0,979 | 0,967 |
| **Time before start of cancer treatment** | 0,649 | 0,689 | 0,789 | 0,056* |
| **Type of healthcare provider** | 0,740 | 0,336 | 0,015* | 0,646 |
| **Type of hospital** | 0,768 | 0,891 | 0,255 | 0,780 |

***** Multilevel analyses were performed for this determinant (p<0,20)
